# Atypical Lung Carcinoid With EML4/ALK Fusion Detected With Circulating Tumor DNA

**DOI:** 10.7759/cureus.22276

**Published:** 2022-02-16

**Authors:** Denise A Gococo-Benore, Ashton Boyle, Natasha Wylie, Leylah Drusbosky, Andras Khoor, Jason S Starr

**Affiliations:** 1 Internal Medicine, Mayo Clinic, Jacksonville, USA; 2 Hematology/Oncology, Mayo Clinic, Jacksonville, USA; 3 Genomics, Guardant Health, Redwood City, USA; 4 Pathology and Laboratory Medicine, Mayo Clinic, Jacksonville, USA

**Keywords:** molecular oncology, neuroendocrine neoplasms, neuroendocrine, ctdna, atypical carcinoid

## Abstract

Atypical carcinoids are a rare subset of neuroendocrine tumors that originate from cells within the bronchopulmonary tree. Compared to typical carcinoids, atypical carcinoids are associated with a worse prognosis. *EML4-ALK* fusions are reported in 5% of non-small cell lung carcinoma, but are rare in atypical carcinoids with only five previously reported cases. We report a case of a 70-year-old female with atypical carcinoid with metastasis to the liver and axial skeleton. She did not respond to standard of care chemotherapy with carboplatin and etoposide and was elected to enroll in hospice because of worsening clinical status. However, a circulating tumor DNA (ctDNA) sample was obtained the same day which revealed an *EML4-ALK* fusion gene. She immediately began therapy with the second-generation ALK inhibitor alectinib, with a remarkable symptomatic and radiographic response. Seven months later, the disease progression was demonstrated in the liver and the patient was switched to the third-generation ALK inhibitor lorlatinib. At the time of writing, the patient has continued to demonstrate sustained clinical, radiographic, and biochemical responses while on lorlatnib for two years. The dramatic treatment results highlighted in this case make the argument to consider ctDNA after the diagnosis of locally advanced or metastatic atypical carcinoid.

## Introduction

Lung carcinoids account for 1-2% of all lung neoplasms of all neuroendocrine tumors (NETs) that occur throughout the body. The grading of these tumors, as typical or atypical, is unique in that mitotic rate and/or necrosis are used without mention of the proliferative index (Ki-67). Typical carcinoids are characterized by a mitotic rate <2 per 10 high-powered fields (HPF) and lack of necrosis. Atypical carcinoids have a mitotic rate of 2 to 10 mitoses per 10 HPF and/or the presence of necrosis. The designation between the two types of lung carcinoids is important in determining the appropriate treatment and prognosis. Typical carcinoids follow a more indolent course and are associated with an excellent prognosis following surgical resection with a rare spread outside of the lung. Atypical carcinoids have a greater tendency to metastasize or recur locally and are associated with a worse prognosis.

Lung carcinoids appear to share the same neuroectodermal progenitor cells as small cell carcinoma and large cell neuroendocrine carcinoma; however, carcinoids of the lung are distinct from non-small cell lung carcinoma (NSCLC) [1,2]. We report a case of atypical carcinoid with identification of an echinoderm microtubule-associated protein-like 4 (*EML4*)/anaplastic lymphoma kinase (*ALK)* fusion first detected with circulating tumor DNA (ctDNA). *EML4/ALK* fusions are reported in 5% of NSCLC and the incidence in atypical carcinoids is rare with only five previously reported cases [3-7].

## Case presentation

A 70-year-old female with no significant past medical history presented to her primary care physician with flank pain. Initial workup with computed tomography (CT) revealed multiple liver and bone lesions. Fluorodeoxyglucose positron emission tomography/computed tomography (FDG PET/CT) revealed lung nodules and liver lesions. Two biopsies of a single liver lesion were performed at an outside facility. Both were read as “low-grade NET,” but only 1% tumor tissue was present for analysis; thus, there was no mention of mitosis, necrosis, or proliferative index (Ki-67). 

**Figure 1:**
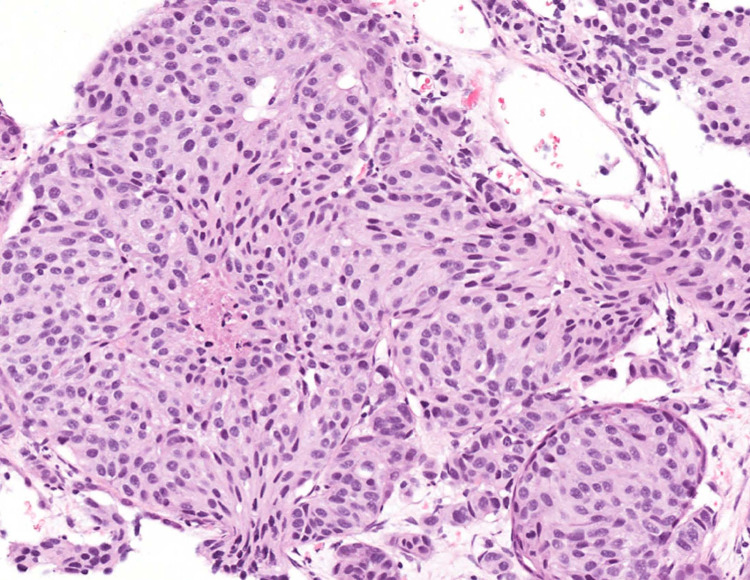
Histologic section in low magnification shows a characteristic organoid growth pattern (hematoxylin-eosin stain, original magnification x130)

**Figure 2:**
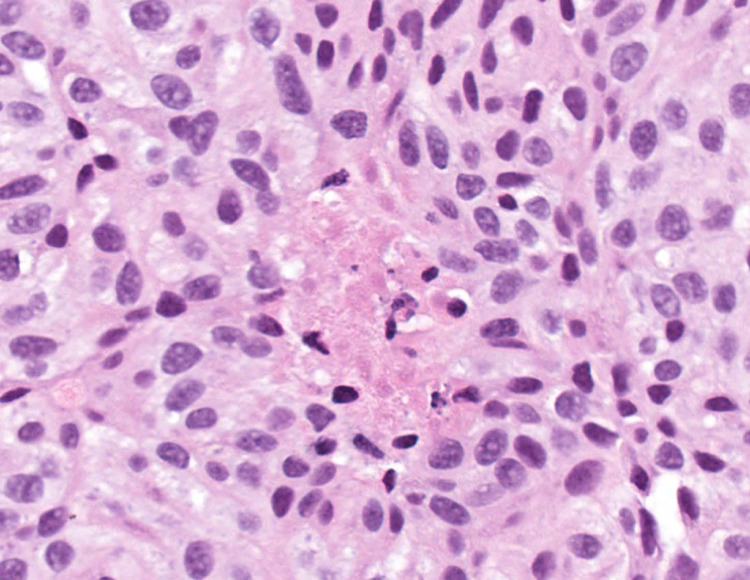
Histologic section in high magnification The histologic section in high magnification reveals uniform tumor cells with finely granular nuclear chromatin and punctate necrosis (center) (hematoxylin-eosin stain, original magnification x400)

At the time of her initial consultation at our facility, the patient complained of fatigue, night sweats, anorexia, and severe back and pelvic pain. Tumor marker evaluation was as follows (normal values included parenthetically): chromogranin 1955 ng/mL (< 93 ng/mL), carcinoembryonic antigen (CEA) 2213.6 ng/ mL (< 2.5 ng/mL), carbohydrate antigen 19-9 (CA19-9) 96 U/mL (<35 U/mL). A CT-guided biopsy of a liver lesion was obtained for diagnostic clarification. Histologic sections showed metastatic carcinoid tumor with characteristic organoid nesting (Figure 1). The tumor cells were uniform in appearance and revealed finely granular nuclear chromatin (Figure 2). The mitotic rate was 3 mitoses per 2 mm^2^ and necrosis was present, consistent with an atypical carcinoid. 

**Figure 3:**
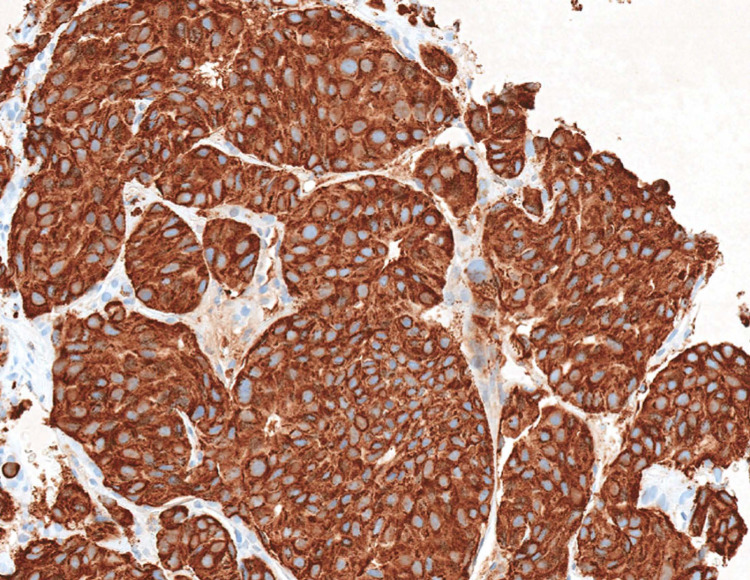
In immunohistochemical studies, the neoplastic cells are strongly positive for chromogranin (original magnification x150)

The immunohistochemical studies showed that the neoplastic cells were positive for chromogranin (Figure 3), synaptophysin, and thyroid transcription factor 1 (TTF-1), supporting the fact that this neoplasm originated in the lung. 

**Figure 4:**
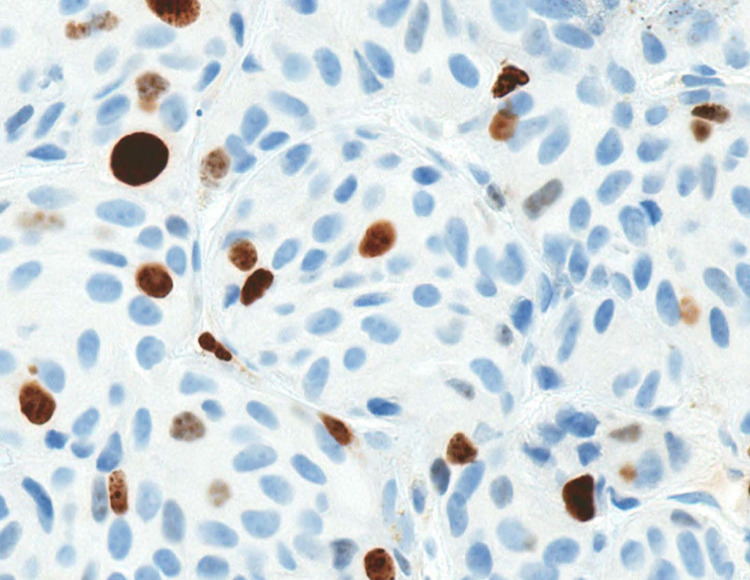
The Ki-67 labeling index is approximately 8% (original magnification x400)

**Figure 5:**
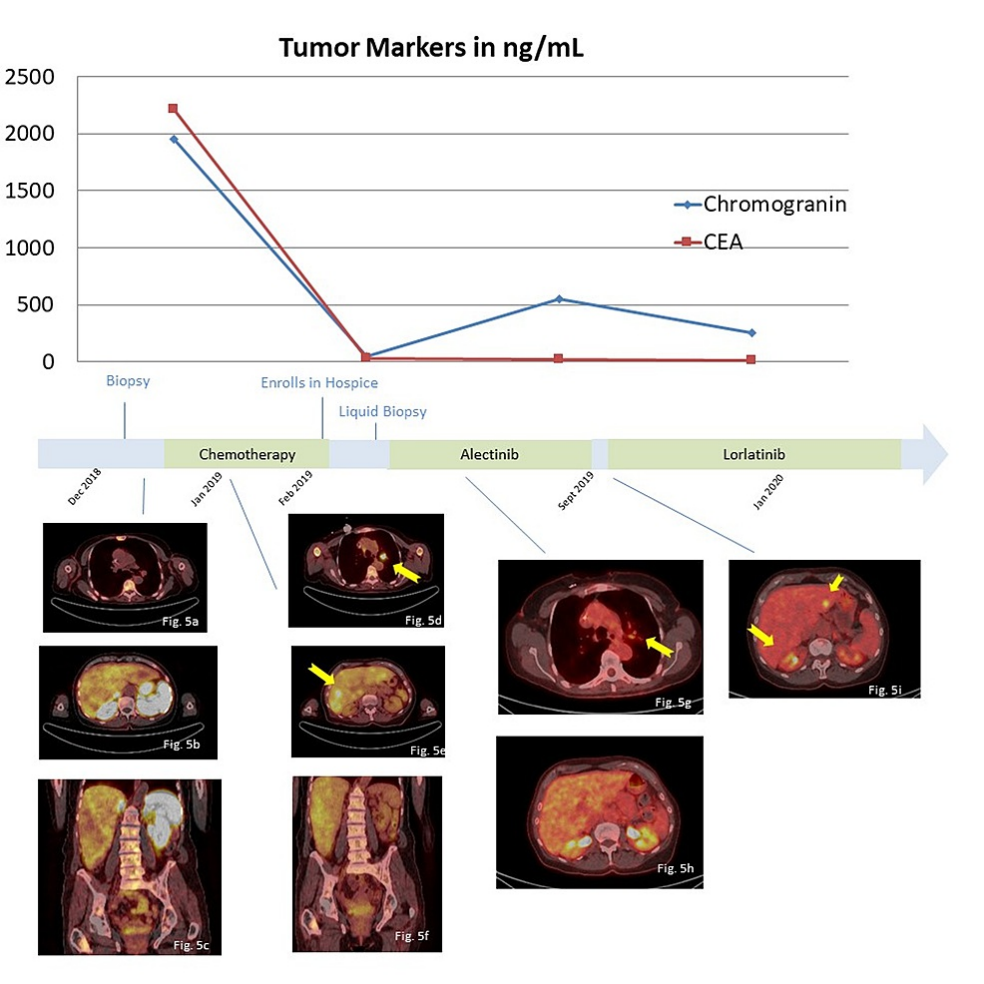
DOTATATE PET/CT scans in done in January 2019 Figures 5a-5c are taken from a DOTATATE PET/CT Scan in January 2019. Figures 5d-5f are images taken from an FDG PET/CT at the same time. There is DOTATATE uptake in the bones (Figure 5c), but minimal uptake in the lungs (Figure 5a) and none in the liver (Figure 5b); however, there are foci of FDG avid disease is seen in the left upper lung nodule (Figure 5d), numerous times in the liver (Figure 5e), and throughout the axial skeleton. This was thought to represent heterogeneity in the disease process. Figures 5g-5h  are FDG PET/CT scans taken in April 2019 during treatment with alectinib demonstrating excellent response. There is decreased uptake in the left upper lobe nodule (Figure 5g). The hepatic lesions seen previously (Figure 5e) have now decreased in size or are no longer seen. Figure 5i was taken from an FDG PET/CT in October 2019 and demonstrates two new foci of FDG activity in the left and right lobes of the liver. This correlates with an interval increase in chromogranin, prompting a switch in therapy to a second-generation ALK inhibitor. PET = Positron emission tomography; CT = Computed tomography; FDG = 18F-fluorodeoxyglucose

Of note, the Ki-67 labeling index was approximately 15% (Figure 4). A Gallium 68 (^68^Ga) DOTATATE (a radiopharmaceutical tracer) positron emission tomography (PET)/CT was also obtained at our institution (Figure 5). Interestingly it showed uptake within the bone and supraclavicular/mediastinal lymph nodes; however, there was minimal or no uptake within the liver or lung lesions.

Chemotherapy was initiated with carboplatin and etoposide. After two cycles, repeat imaging showed a clear progression of the disease. The patient’s performance status was also declining quickly. Given the lack of benefit of the cytotoxic chemotherapy and her eastern cooperative group (ECOG) performance status of 3, the patient was enrolled in hospice. Before the patient left the office that day, circulating tumor DNA (ctDNA) was obtained for the rare chance that a targetable mutation could be identified. One week later, Guardant360 testing (Guardant Health Inc., Redwood City, USA) revealed an *EML4-ALK* fusion gene with a variant allele fraction of 6.1%. The fusion breakpoints identified by liquid biopsy were *EML4*(2:42505369)-*ALK*(2:29447480); where *EML4 *is the upstream gene (N-terminus). The patient was called immediately and opted to start on the second-generation ALK inhibitor alectinib. The patient was able to receive the medication in two days. One week later, the patient presented for follow-up and exhibited dramatic improvement in energy, appetite, and pain. Additionally, the patient’s dyspnea had resolved. After two months of therapy, the 18F-fluorodeoxyglucose (FDG) PET/CT imaging demonstrated dramatic response with shrinkage of two pulmonary nodules, a decrease in the size of numerous hepatic metastasis, interval resolution of lymphadenopathy within the neck, and sclerotic lesions in the axial skeleton consistent with treatment response (Figure 5). Labs at the time demonstrated decreasing tumor markers (chromogranin 44 ng/mL, CEA 27.1 ng/mL). Of note, the patient’s tissue was also sent for next-generation sequencing, which confirmed the presence of the *EML4-ALK* fusion gene.

Seven months into the treatment with alectinib, FDG PET/CT demonstrated progression of disease in the liver (Figure 5). Tumor markers were also increasing (chromogranin 552 ng/mL and CEA 19.6 ng/mL). Repeat liquid biopsy identified *ALK* G1202R mutation (an acquired resistance alteration to alectinib) with a variant allele fraction of 1.9%. The patient was then started on a third-generation ALK inhibitor, lorlatinib, which has been shown to overcome this resistance alteration. The patient initially experienced side effects including word-finding difficulties, vivid dreams, personality changes, and peripheral neuropathy. A dose reduction was performed with improvement in the side effects. The patient has continued to experience sustained clinical, radiographic, and biochemical responses while on lorlatinib [8]. It has been two years now since the patient was disenrolled in hospice.

## Discussion

Anaplastic lymphoma kinase (ALK) rearrangements encode oncogenic fusion proteins that ultimately result in unregulated cellular growth and proliferation. Over 20 fusion partners of *ALK* have been identified with *EML4* being the most common [9]. The *EML4-ALK* fusion gene has been identified in approximately 5% of all-comers with NSCLC. The fusion protein has been described almost exclusively with adenocarcinoma histology and is much more common in light or never smokers. The finding of *EML4/ALK* in lung carcinoids is exceedingly rare [10]. A retrospective analysis of 462 lung carcinoid patients in the Guardant Health database found at least one somatic alteration detected in 89% of patients, with the most common alterations occurring in *TP53* (55%), *EGFR *(21%), *KRAS* (17%), *ATM* (16%), *PIK3CA* (15%), *ARID1A* (12%), *BRAF* (11%), *RB1* (11%), and *APC* (9%), with EML4-ALK fusions detected in 1.5% of patients. To our knowledge, there have only been five other reports of ALK rearrangements detected in atypical carcinoids [3-5]. According to cBioPortal for cancer genomics (www.cbioportal.org), the most common molecular abnormalities in atypical lung carcinoid tumors occur in *MEN1* (31%), *EIF1AX* (23%), *SF3B1* (15%), *MST1R* (10%), *FYN* (10%), *ARID1A* (8%), *RAF1* (8%), and *ATM* (8%), though analyzed in a very limited sample size (N=13).

There are several available methods that are currently employed for detecting the *ALK* fusion gene, all of which have their benefits and pitfalls. Fluorescence in situ hybridization (FISH), immunohistochemical staining (IHC), and reverse transcriptase-polymerase chain reaction (RT-PCR) are all commonly performed on tissue samples. However, each share concerns intrinsic to tissue biopsy sampling. IHC and FISH depend on the interpretation of the pathologist, and both false positives and false negatives can result accordingly. RT-PCR provides more objective results, but not all *EML4-ALK* variants can be detected as specific primer pairs for *ALK* rearrangements are required [9]. Next-generation sequencing (NGS) rectifies the latter issue, as a single analysis on a sample can provide many mutations without having to predict the presence of a mutation beforehand [9]. Tissue biopsies are not only invasive and difficult on the patient, but also risk missing any mutational heterogeneity of cancer tissue within a single tumor or between metastatic sites [11].

CtDNA is being increasingly used, especially in conjunction with tissue NGS, to detect mutations or fusions in a variety of different solid tumor cancers. Liquid biopsy has many advantages over tissue biopsy. It is less invasive than tissue biopsies and thus can be repeated whenever necessary. It can also provide a dynamic biomarker to help to determine the efficacy of a therapy. In this rapidly declining patient, time was of the essence to find an actionable target. Circulating tumor DNA results typically report out within one week, while tissue NGS can take up to 2-3 weeks to get results. Circulating tumor DNA has been demonstrated to identify potentially actionable mutations in neuroendocrine tumors, thus guiding therapy; however, it is not routinely utilized in clinical practice [12].

In addition to the diagnostic ability of ctDNA, it can also be used to provide information about mechanisms of resistance to targeted therapies [11,13]. This may be particularly useful when treating tumors with *ALK* rearrangements, as all FDA-approved first-and second-generation ALK inhibitors are known to acquire resistance and lose treatment efficacy over time [14].

## Conclusions

We report a rare case of an *EML4-ALK*-positive pulmonary atypical carcinoid that has responded for over two years to ALK inhibitors. This case contributes to the scarce literature of *ALK* rearrangements in atypical carcinoids. The positive results of the case we have presented make the argument for considering ctDNA after the diagnosis of locally advanced or metastatic atypical carcinoid. Given the lack of widespread use of NGS in atypical carcinoid the true incidence of *ALK* rearrangements is not known. Further research should be conducted to establish the epidemiology of *ALK* rearrangements in atypical carcinoids and better determine the utility of ctDNA testing to guide therapy in this diagnosis.
